# Posterior to anterior migration of a large optic fragment in a pseudophakic eye

**DOI:** 10.3205/oc000133

**Published:** 2020-02-27

**Authors:** Sidney A. Schechet, Seenu M. Hariprasad, Kamran Riaz

**Affiliations:** 1 The University of Chicago, Department of Ophthalmology and Visual Sciences, Chicago, United States

**Keywords:** anterior chamber, intraocular lens, dislocated lens, IOL, pupilloplasty

## Abstract

**Objective:** To report a curious case of a posteriorly dislocated large optic fragment that migrated anteriorly to the anterior chamber through a well-positioned scleral-fixated intraocular lens (SFIOL) and intact pupil.

**Methods:** Single case report.

**Results:** The large optic fragment was removed successfully in the operating room.

**Conclusions:** While eyes with a posteriorly-displaced lens or IOL remnants may occasionally be observed without intervention, these patients should be monitored closely.

## Introduction

There are a few reports that have shown posterior to anterior migration of large Ozurdex implants in pseudophakic eyes [1], [2], [3]. Herein, we present an interesting case of a large IOL optic fragment that migrated anteriorly in a pseudophakic eye and intact pupil.

## Case description

**Figure 1:**
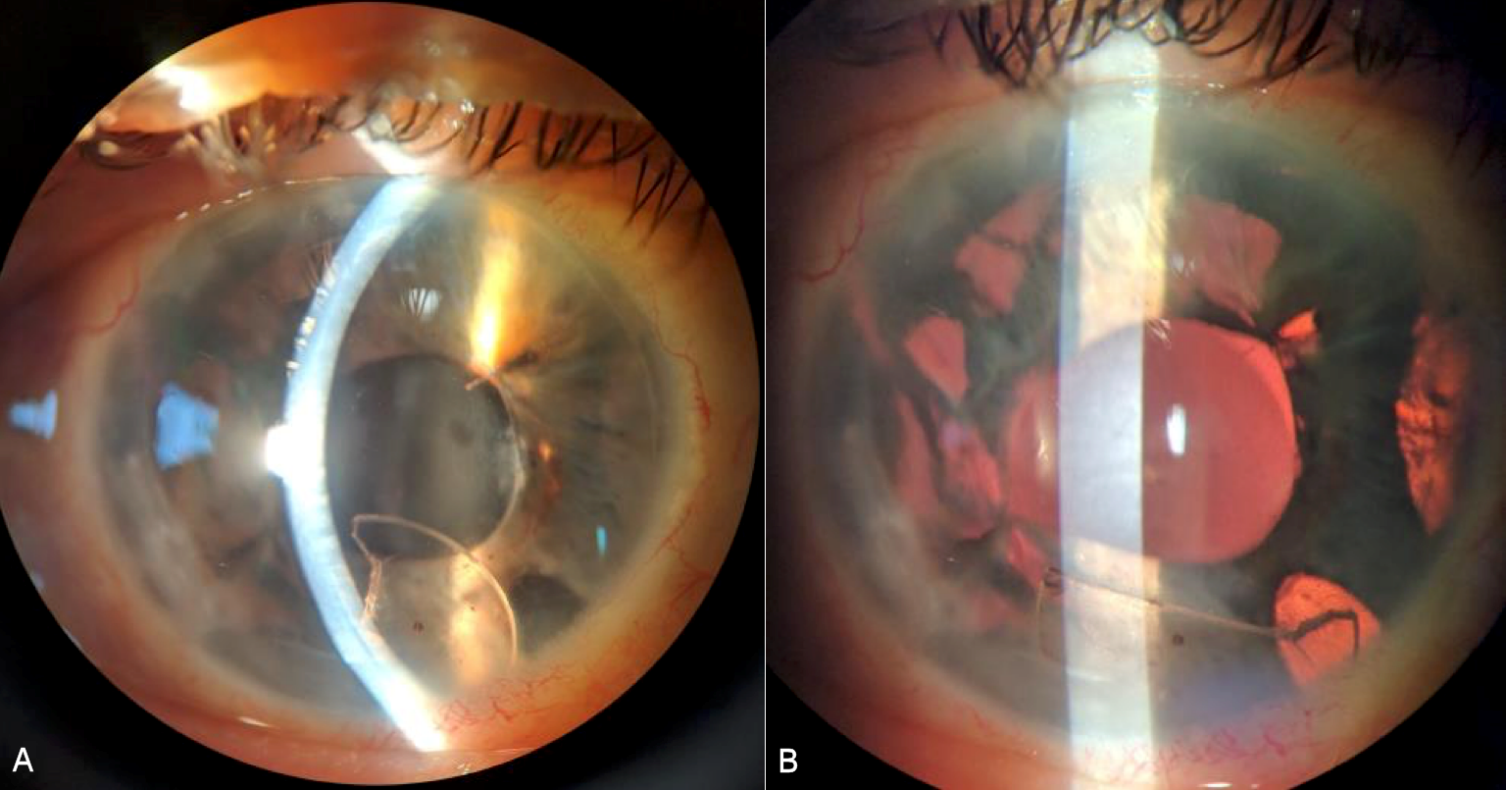
A) Anterior segment photograph of large optic fragment (arrow) in the anterior chamber with mild corneal edema. B) Same image with retroillumination highlighting the iris transillumination defects.

A 64-year-old male had multiple right eye surgeries including vitrectomy, lensectomy, and scleral-buckling for retinal detachment and mature cataract, and an anterior chamber intraocular lens (ACIOL) insertion for aphakia. The ACIOL was a Kelman style multiflex III single-piece PMMA material (MTA4UO; Alcon Laboratories, Fort Worth, TX USA). In the few months after ACIOL insertion, the patient developed a severe corneal edema that was unresponsive to medical therapy, so the patient underwent another surgery to remove the ACIOL. The ACIOL was cut in 3 locations: at the two haptic-optic junctions and through the entire optic using MST 23G micro-holding forceps and 19G Packer/Chang IOL cutters (MST Technologies, Redmond, WA USA). The surgical decision was made to cut the ACIOL into 4 pieces in order to facilitate the removal through a 2.75 mm primary clear corneal incision placed superiorly. The primary surgeon determined that in order to explant the ACIOL in one piece, a large incision, including but not limited to a scleral tunnel incision, would have been necessary and this would provide a surgical disadvantage for anterior chamber stability during scleral fixation portion of the new posterior chamber IOL. However, intraoperatively, one ACIOL piece (around 45% of the optic) fell posteriorly, but the surgery continued where a SFIOL was placed and pupilloplasty performed with no issues. Postoperatively, the cornea cleared, the SFIOL was in perfect position, and the pupilloplasty was intact and round measuring 4.5 mm in diameter. The dilated exam was unremarkable with the optic fragment seen inferiorly without retinal tears or detachment, and the patient elected for observation. Six weeks postoperatively, the patient presented with new glimmering in this right eye after sneezing and coughing. Slit-lamp examination revealed that the large ACIOL optic piece had migrated into the anterior chamber (Figure 1A (Fig. 1)) despite having a well-positioned SFIOL and pupilloplasty sutures fixing the iris diameter at 4.5 mm. The optic fragment was removed successfully in the operating room. 

## Discussion

Previous reports have shown posterior to anterior migration of large Ozurdex implants in pseudophakic eyes [1], [2], [3]. Similarly, we describe a larger-sized IOL optic fragment that migrated anteriorly. The pupil was round and fixed to 4.5 millimeters in diameter after a pupilloplasty – and while there was an inferior iridectomy and multiple iris transillumination defects (Figure 1B (Fig. 1)), the optic piece was larger in size. The three-piece SFIOL was well-centered and positioned nicely in the sulcus space. The patient noted vision changes in this eye immediately after having suddenly sneezing and coughing, so the optic piece may have slipped past the SFIOL and through the pupil at that time. Timely surgical intervention with optic removal via anterior approach prevented corneal decompensation.

## Conclusion

To the authors knowledge, there are no reports of anterior migration of optic fragments in pseudophakic eyes. While eyes with a posteriorly-displaced lens or IOL remnants may occasionally be observed without intervention, these patients should be monitored closely.

## Notes

### Competing interests

The authors declare that they have no competing interests.
